# Evaluation of methylene blue solution distribution in the four-point transversus abdominis plane block technique in pigs: a pilot anatomical study

**DOI:** 10.3389/fvets.2025.1574833

**Published:** 2025-04-16

**Authors:** Jerneja Sredenšek, Jana Brankovič, Urša Lampreht Tratar, Maja Čemažar, Mihajlo Đokić, Alenka Seliškar

**Affiliations:** ^1^Small Animal Clinic, Veterinary Faculty, University of Ljubljana, Ljubljana, Slovenia; ^2^Institute of Preclinical Sciences, Veterinary Faculty, University of Ljubljana, Ljubljana, Slovenia; ^3^Department of Experimental Oncology, Institute of Oncology Ljubljana, Ljubljana, Slovenia; ^4^Faculty of Health Sciences, University of Primorska, Izola, Slovenia; ^5^Faculty of Medicine, University of Ljubljana, Ljubljana, Slovenia; ^6^Department of Abdominal Surgery, University Medical Centre Ljubljana, Ljubljana, Slovenia

**Keywords:** abdominal surgery, cadaveric anatomical study, pig, regional anaesthesia, transversus abdominis plane block, methylene blue

## Abstract

**Aim:**

This prospective pilot anatomical study aimed to develop an ultrasound-guided transversus abdominis plane (TAP) block technique that desensitises cranial and mid-abdominal wall in grower pigs. We hypothesised that a four-point TAP approach would be more efficient than a three-point TAP in staining relevant nerves of the cranial and mid-abdominal wall.

**Methods:**

In phase I, the ultrasound anatomy of the abdominal wall musculature was examined on three pig cadavers (two piglets and one fattening pig) and the ultrasound localization of the needle in the corresponding interfascial plane was practised. In phase II, a three-point TAP injection was performed in three freshly euthanized cadavers of grower pigs. A 1% methylene blue solution (0.3 mL/kg per injection point) was injected between the transversus abdominis and internal oblique muscle. In phase III, methylene blue solution was injected at four points (0.2 mL/kg per injection point) in four anaesthetized grower pigs prior to euthanasia. Positive nerve staining was defined as continuous staining of at least 1 cm of the nerve length. Binary variables (positive/negative) were used for nerve staining assessment.

**Results:**

The four-point TAP technique with a lower injection volume stained more nerves than the three-point technique with a higher injection volume, i.e., 69% of the observed nerves from the eighth-last thoracic to the third lumbar nerve were stained with the four-point TAP technique. The nerves in the centre were stained with a higher success rate, while the eighth-last thoracic and the second lumbar nerve were stained with less success (1/8 and 3/8, respectively). The third lumbar nerve was not stained.

**Conclusion:**

The four-point TAP technique could be used as part of a multimodal analgesia approach for cranial and mid-abdominal surgery in pigs, but live animal studies are needed to evaluate the clinical applicability and efficacy of desensitisation.

## 1 Introduction

The transversus abdominis plane (TAP) block is an interfascial plane block. Local anaesthetic is deposited in the plane between the internal oblique (IOM) and transversus abdominis (TAM) muscles to block the ventral branches of the thoracolumbar spinal nerves (1, 2). In animals, the TAP block is used to desensitise tissue structures of the ventral and lateral abdominal wall, including the parietal peritoneum, abdominal muscles and subcutaneous tissue as well as the mammary glands. An improvement in pain scores in visceral pain due to abdominal surgery or pancreatitis has also been described (3).

Different techniques and approaches have been described in domestic (4–12) and wild (13) animal species. The one-point TAP technique is described in cats (5), rabbits (7) and Canadian lynx (13), the two-point technique in dogs (4, 10), cats (9), pigs (11, 12) and horses (8), while the three-point technique is described in cats (9), pigs (12), and ponies (6).

The abdominal wall in the cranial abdominal region of pigs (usually with 15–16 thoracic vertebrae) is innervated by the last half of the thoracic nerves, which regionally accompany the ribs in the thoracic wall and enter the abdominal wall ventrally. In the middle abdominal region, innervation is mainly provided by the first three to four lumbar nerves, which emerge from the lumbosacral plexus and have specific names; the first ventral branch (L1) is called the iliohypogastric nerve, the second (L2) is called the ilioinguinal nerve, the third (L3) and fourth (L4) unite to form the genitofemoral nerve, which runs caudally and supplies the skin of the thigh and genitals (14).

The two-point TAP technique in pigs (11) with caudal retrocostal and lateral approaches resulted in successfully staining a median (range) of 5 (4–7) target nerves from the fourth-last thoracic nerve to the second lumbar nerve innervating the periumbilical and caudal abdominal wall. Only moderate success has been achieved with another two- or three-point technique (12). Injections were made cranially at two thirds of the distance between the xyphoid process and the iliac crest, ventral to the rib arch, and caudally ventral to the last rib (two-point technique). In the three-point technique, an additional injection was made halfway between the cranial and caudal injection points.

Some surgical procedures in the epigastrium require an incision up to the level of the xyphoid process (15), and the TAP techniques already described in pigs (11, 12) do not enable complete desensitisation of the cranial portions of abdominal wall. This pilot study is therefore set out to (1) delineate a TAP approach targeting nerve branches responsible for the somatosensory supply of cranial and mid-abdominal wall in pigs, and (2) evaluate the distribution patterns and nerve involvement resulting from TAP injections using either three- or four-point approaches with clinically applicable volumes of dye solution in pig cadavers and anaesthetized pigs, respectively. We hypothesised that a four-point TAP injection approach would be required to reach all relevant nerves of the cranial and mid-abdominal wall.

## 2 Materials and methods

### 2.1 Experimental design

**Figure 1:**
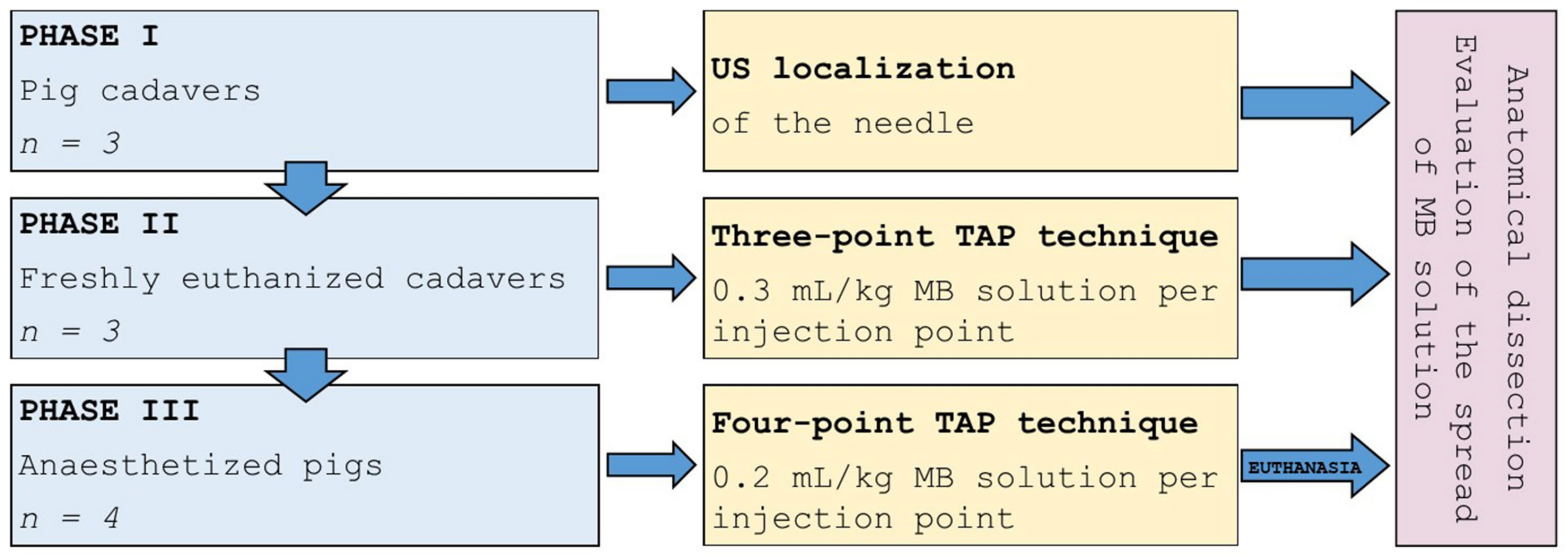
Study design. The study was conducted in three phases; cadavers were used for the first and second phases and anaesthetized pigs were used in the third phase. MB, methylene blue; US, ultrasound.

This pilot study was conducted in three phases (Figure 1). The first phase served to familiarise with the anatomy of the abdominal wall in pig cadavers and to practise the ultrasound (US) localisation of the needle in the appropriate interfascial plane between the IOM or its aponeurosis and the TAM. The cadavers of pigs (*n =* 3) deceased for reasons unrelated to infectious diseases and kept in the cold room for several days before being used in the study were obtained from the Veterinary Hygiene Service.

Next, the distribution of a methylene blue (MB) solution in a three-point TAP technique was examined in freshly euthanized cadavers (phase II, *n* = 3) within 1 h after euthanasia. The abdominal organs were removed from cadavers by midline laparotomy based on the requirements of the primary study (16) and the abdominal wall was sutured. Then the TAP study was performed.

Finally, the distribution of MB solution was studied using a four-point TAP technique performed in anaesthetized pigs 15 min prior to euthanasia (phase III, *n* = 4). After that, the abdominal organs were removed from cadavers by midline laparotomy for the primary study. The abdominal wall was sutured before anatomical dissection was performed to assess the spread of MB solution.

### 2.2 Animals

Phases II and III were approved by the National Ethics Committee and National Veterinary Administration (approval number U34401-2/2021/5, approval date 3.3.2021) and conducted in accordance with European Directive 2010/63/EU, ARRIVE 2.0 guidelines (17), and PREPARE guidelines for planning animal research and testing (18). The TAP study was designed in compliance with the 3Rs principle (Replacement, Reduction, Refinement) in addition to the primary study investigating the effects and safety of electroporation with bleomycin on liver vessels (16).

Pigs (*Sus scrofa domesticus*), six female and one castrated male Landrace and Large White hybrids, aged 10 weeks, were procured from a local farm free of swine fever, Aujeszky’s disease, porcine respiratory and reproductive syndrome, and salmonellosis. They were vaccinated against mycoplasmosis 4–6 weeks before purchase. During 2 weeks of acclimatisation and habituation, they were housed in three groups (two pairs and one group of three pigs). After surgery (primary study), they were housed in separate indoor straw-bedded pens that enabled visual, auditory, and limited tactile contact. Seven days later, they were returned to the initial groups. They were exposed to a natural light–dark cycle with an ambient temperature of 19 to 21°C. Besides the straw bedding, various methods of environmental enrichment, such as plastic balls with openings for treats and hiding fruit and mealworms in the straw, were used. The pigs were fed a commercial pig diet twice daily, and water was provided *ad libitum* from nipple waterers. They were under continuous video surveillance and clinically examined three times daily until the end of experiment.

Each pig was anaesthetized twice for the purpose of the primary study (16). They were first anaesthetized for a median laparotomy for resection and electroporation of the portal vein and adjacent tissues. Four weeks later, when the TAP study was carried out, they were anaesthetized again and euthanized with T61 (MSD, Intervet International B.V., Kenilworth, NJ, USA) 3 mL/10 kg intravenously.

### 2.3 The TAP block technique

The TAP injections were performed by two anaesthetists (JS and AS) previously trained in performing TAP blocks in dogs but not in pigs. The pigs were positioned in dorsal recumbency with the hindquarters turned slightly to the left or right. Injection sites were determined using anatomical landmarks and ultrasonography. An US device (M9 GI, Mindray, Nanshan, Shenzhen, China) with a linear 3.0–13.0 MHz transducer, always placed with the indicator oriented dorsally, was used. Sterile 0.9% NaCl solution (B Braun, Melsungen, Germany) was used to facilitate acoustic coupling. An 8 cm long 22-gauge echogenic needle (Stimuplex Ultra, B Braun, Melsungen, Germany) was advanced in-plane from the ventral to dorsal direction at an angle of 20–30° relative to the skin. A 1% MB solution (1% Metilensko modrilo, UKC, Slovenia) at a dose of 1 mg/kg was diluted with 5% glucose solution (B Braun, Melsungen, Germany) to the calculated volume and used as a dye. A total of 1.8 mL/kg (0.3 mL/kg per injection point) MB solution was used for the three-point TAP technique in phase II, and a total of 1.6 mL/kg (0.2 mL/kg per injection point) MB solution was used for the four-point TAP technique in phase III. When the tip of the needle was visualised in the interfascial plane, 1 mL of the MB solution was injected as part of the total amount available to confirm correct positioning before the entire dose was administered. If the positioning was incorrect, the needle was repositioned, and the procedure was repeated.

### 2.4 The three-point TAP technique: injection points

First injection point: An orientation line between the caudal end of the xyphoid process and the most caudal end of the last rib’s body was drawn and divided into thirds. The US transducer was placed perpendicular to the spine between the second and the last third of the orientation line, just below the costal arch. The TAM and IOM aponeurosis were identified, and MB solution was injected between their fasciae.

Second injection point: A line perpendicular to the spine was drawn through the most caudal end of the last rib’s body. The US transducer was placed on this line at the halfway between the linea alba and the spine and slowly pushed dorsally to find the point where the aponeurosis of the TAM transforms into its muscle part. The MB solution was injected interfascially between the TAM and the IOM aponeurosis just before it transforms into its muscle part.

Third injection point: A line parallel to the spine was drawn through the ischial tuberosity. Two lines perpendicular to the spine were drawn, with the first passing through the most caudal end of the last rib’s body and the second cranial to the coxal tuberosity. The transducer was placed halfway between the intersection of perpendicular lines and a line parallel to the spine. The TAM and IOM were identified, and MB solution was injected in the interfascial plane, approximately at the same dorsoventral level as the second injection point.

### 2.5 The four-point TAP technique: injection points

**Figure 2:**
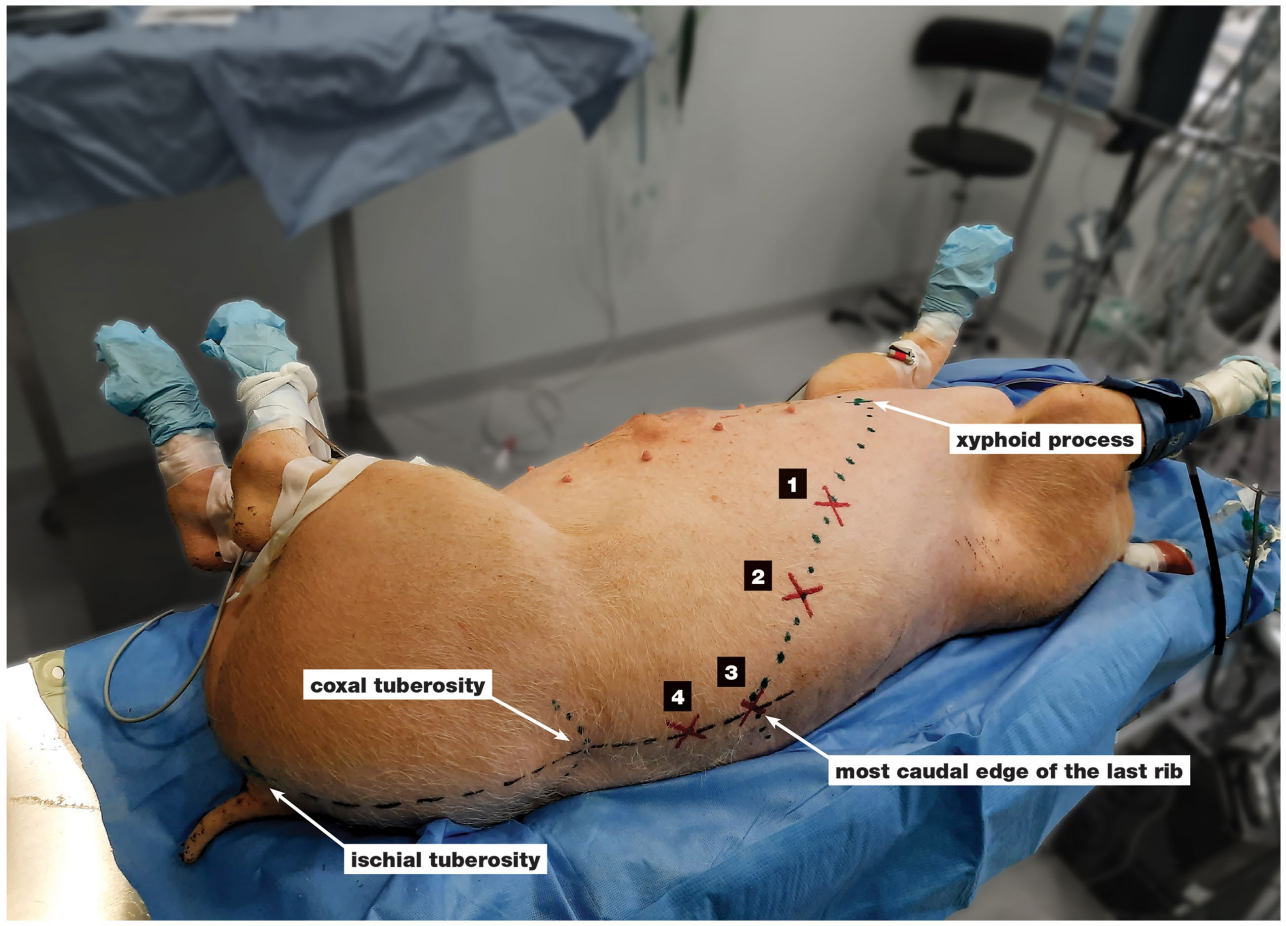
Landmarks of the four-point TAP technique in the anaesthetized pig. 1 half of the orientation line between the xyphoid process and the caudal end of the last rib’s body, 2 three quarters of the orientation line between the xyphoid process and the caudal end of the last rib’s body, 3 the caudal end of the last rib’s body, 4 a line parallel to the spine was drawn through the ischial tuberosity and intersected at the halfway between the caudal end of the last rib’s body and the coxal tuberosity.

**Figure 3:**
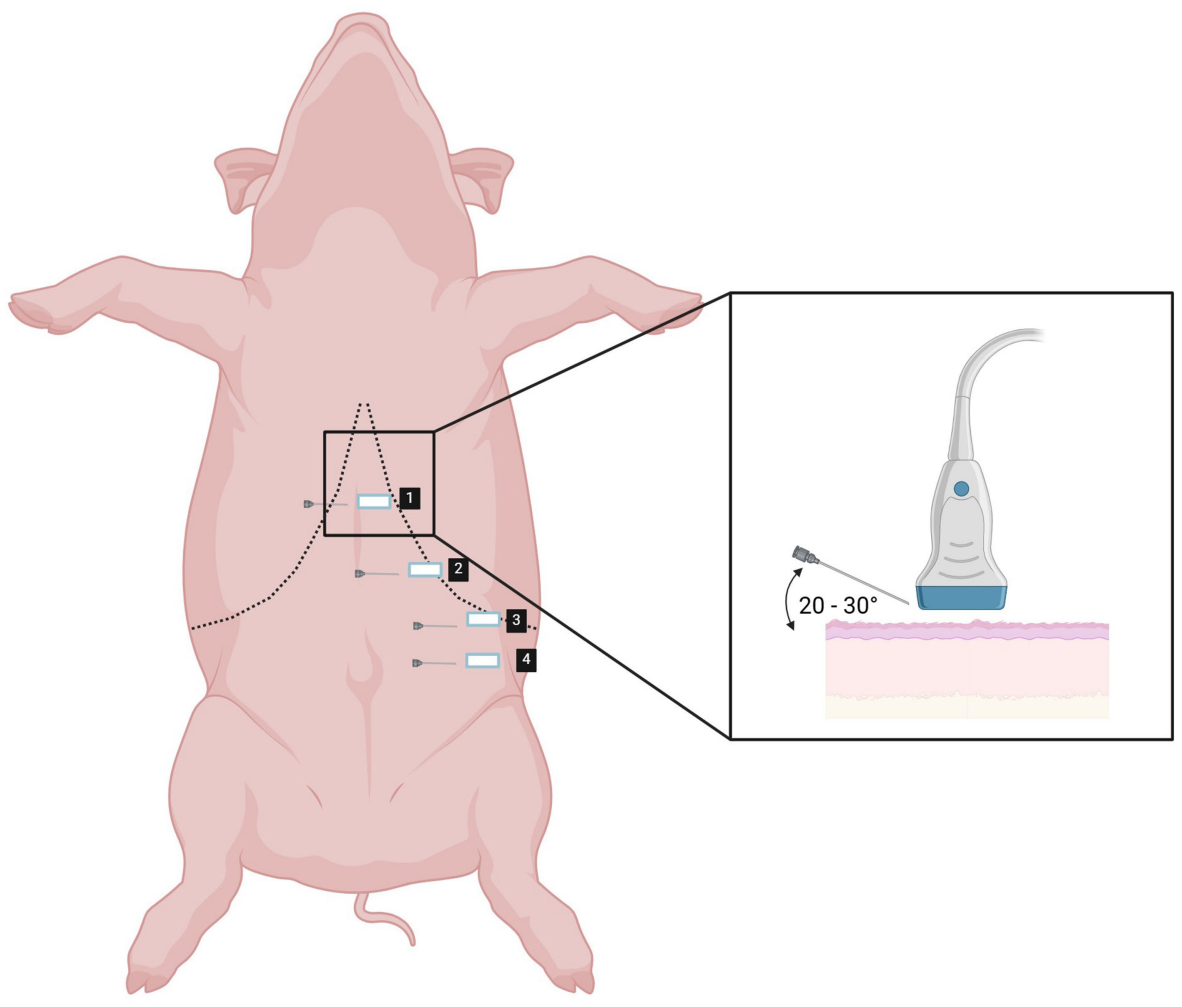
Schematic representation of landmarks (1–4) and the position of the US transducer and its relationship to the needle for performing four-point TAP technique. Created in BioRender. Cemayar, M. (2025) https://BioRender.com/k14z188.

The landmarks of the four-point TAP technique in anaesthetized pig are shown in Figure 2. The schematic representation of landmarks as well as the position of the US transducer and its relationship to the needle can be seen in Figure 3. For the first and second injection points, the orientation line was drawn between the caudal end of the xyphoid process and the most caudal end of the body of the last rib.

**Figure 4:**
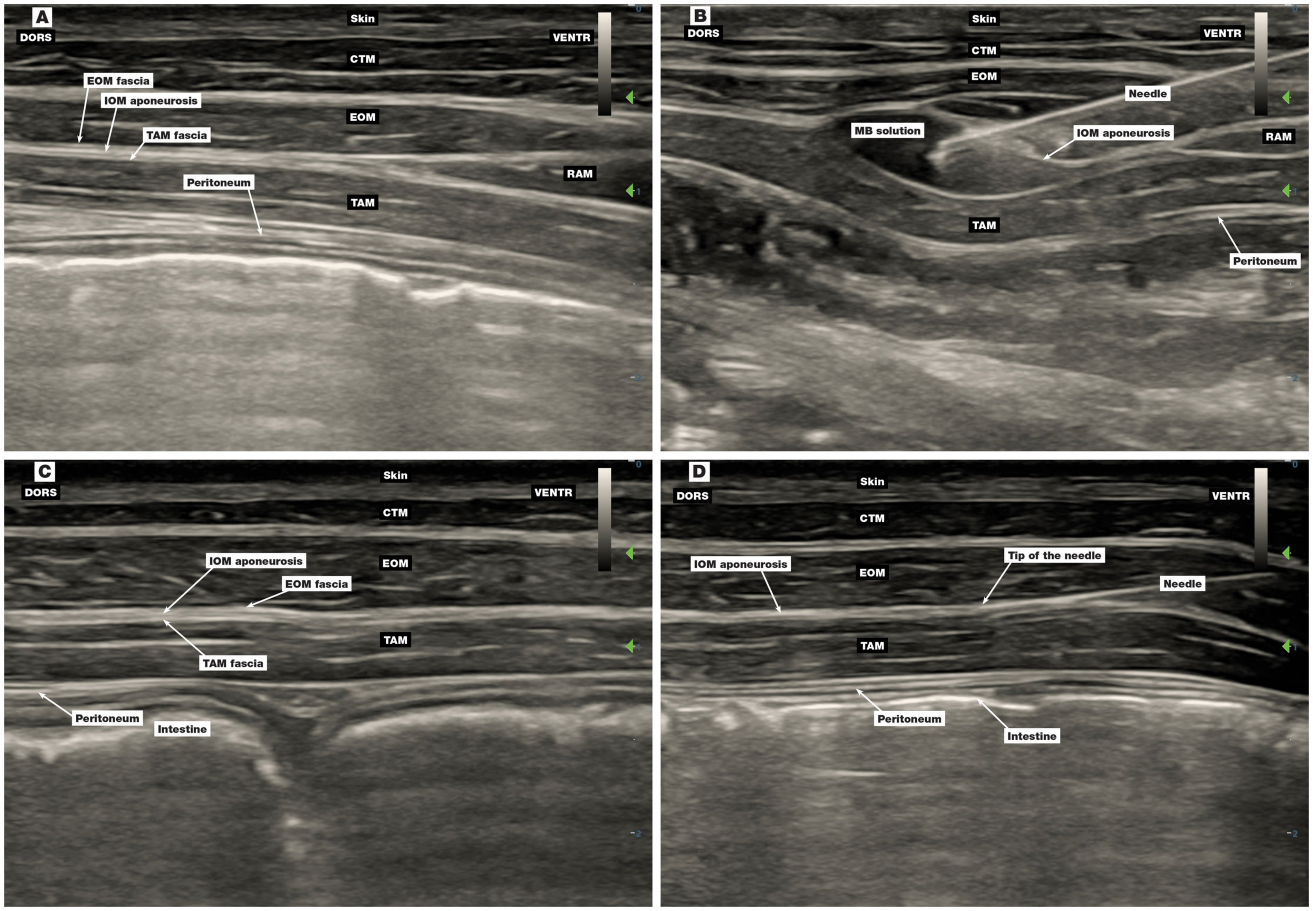
Ultrasound image of the first and second injection points of the four-point TAP block technique. **(A,B)** The first injection point, **(C,D)** the second injection point, **(B)** injection of MB solution between the IOM aponeurosis and TAM fascia, **(D)** position of the needle before injection of MB solution between the IOM aponeurosis and TAM fascia. CTM, cutaneous trunci muscle; DORS, dorsal; EOM, external oblique abdominis muscle; IOM, internal oblique abdominis muscle; MB, methylene blue; RAM, rectus abdominis muscle; TAM, transversus abdominis muscle; VENTR, ventral.

First injection point: The US transducer was placed perpendicular to the spine below the costal arch on a halfway of the orientation line. The rectus abdominis muscle (RAM) was identified ventrally and, with the transducer sliding dorsally towards the costal arch, the IOM aponeurosis and underlying TAM were identified. The MB solution was injected between the fasciae of the latter two structures (Figures 4A,B).

Second injection point: The US transducer was placed perpendicular to the spine below the costal arch in a three-quarter orientation line from cranial to caudal direction. The MB solution was injected interfascially between the TAM and IOM aponeurosis (Figures 4C,D).

**Figure 5:**
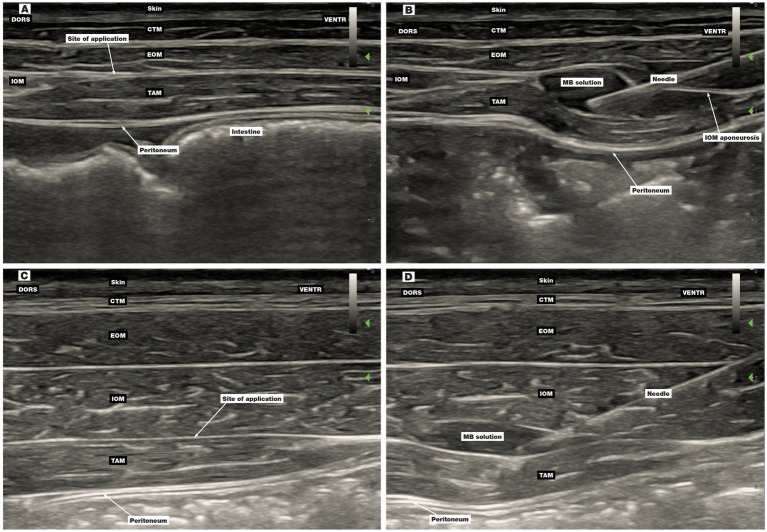
Ultrasound image of the third and fourth injection points of the four-point TAP block technique. **(A,B)** The third injection point, **(C,D)** the fourth injection point, **(B)** injection of MB solution between the IOM aponeurosis and TAM fascia, **(D)** injection of MB solution between the IOM and TAM fascia. CTM, cutaneous trunci muscle; DORS, dorsal; EOM, external oblique abdominis muscle; IOM, internal oblique abdominis muscle; MB, methylene blue; RAM, rectus abdominis muscle; TAM, transversus abdominis muscle; VENTR, ventral.

The third (Figures 5A,B) and fourth (Figures 5C,D) injection points were the same as the second and the third injection points in phase II.

### 2.6 Anatomical dissection and evaluation of the spread of MB solution

All animals were dissected by a veterinary anatomist (JB) with few modifications of previous reports (11, 12). A sagittal incision at both hemiabdomens was made through the skin medially to the teats because the ventral midline was previously incised and sutured for the purpose of the primary study. Further incisions were made perpendicular to the sagittal incision up to the dorsal midline between the xyphoid cartilage and the coxal tuberosity, removing the skin, the cutaneous trunci, and the latissimus dorsi muscles. Other layers, including the external oblique muscle (EOM), IOM, RAM, and TAM were dissected layer by layer. Special attention was given to the dissection of the IOM aponeurosis to reveal the layer where the ventral branches of the thoracic and lumbar nerves run. The RAM was dissected from the abdominal wall to better define the course of the caudal thoracic and the cranial lumbar nerves that run slightly caudoventrally and supply the abdominal wall muscles.

Thoracic nerves were identified according to the number of the ribs in each animal together with the position of the nerves relative to the last rib. The ventral branch of the last thoracic nerve, the costoabdominal nerve, was identified as the nerve running caudally to the last asternal rib. Other thoracic nerves were identified and named in a caudocranial direction as reported previously (11), including the second-last, third-last, fourth-last, etc. The first three lumbar nerves were dissected and identified according to their cranio-caudal position (14).

Since the MB colouration of the tissue develops upon exposure to the air/oxygen (19), the evaluation of staining was performed 5 min after the removal of the IOM from the TAM. The MB staining of the nerves was evaluated through direct visualisation by the anatomist and both anaesthetists. A positive nerve staining was defined as more than 1 cm of continuous staining of the nerve length (11, 12). After evaluating the spread of MB solution in one hemiabdomen, the entire procedure was repeated on the other side. The number of ribs was determined at the end of the evaluation.

### 2.7 Statistical analysis

Binary variables (positive/negative) were used for nerve staining assessment. The average number of stained nerves and the proportion relative to all studied nerves were calculated. The weight of pigs in phase II and III is presented as mean ± standard deviation. Data were analysed using SPSS software (ver. 28, SPSS Inc., IL, USA).

## 3 Results

Pig cadavers in phase I weighed 6, 14, and 70 kg. The pigs weighed 45.7 ± 3.2 kg and 40.0 ± 9.2 kg in phase II and III, respectively. The MB solution effectively stained the tissues and enabled proper dye distribution evaluation.

### 3.1 Phase I

**Figure 6:**
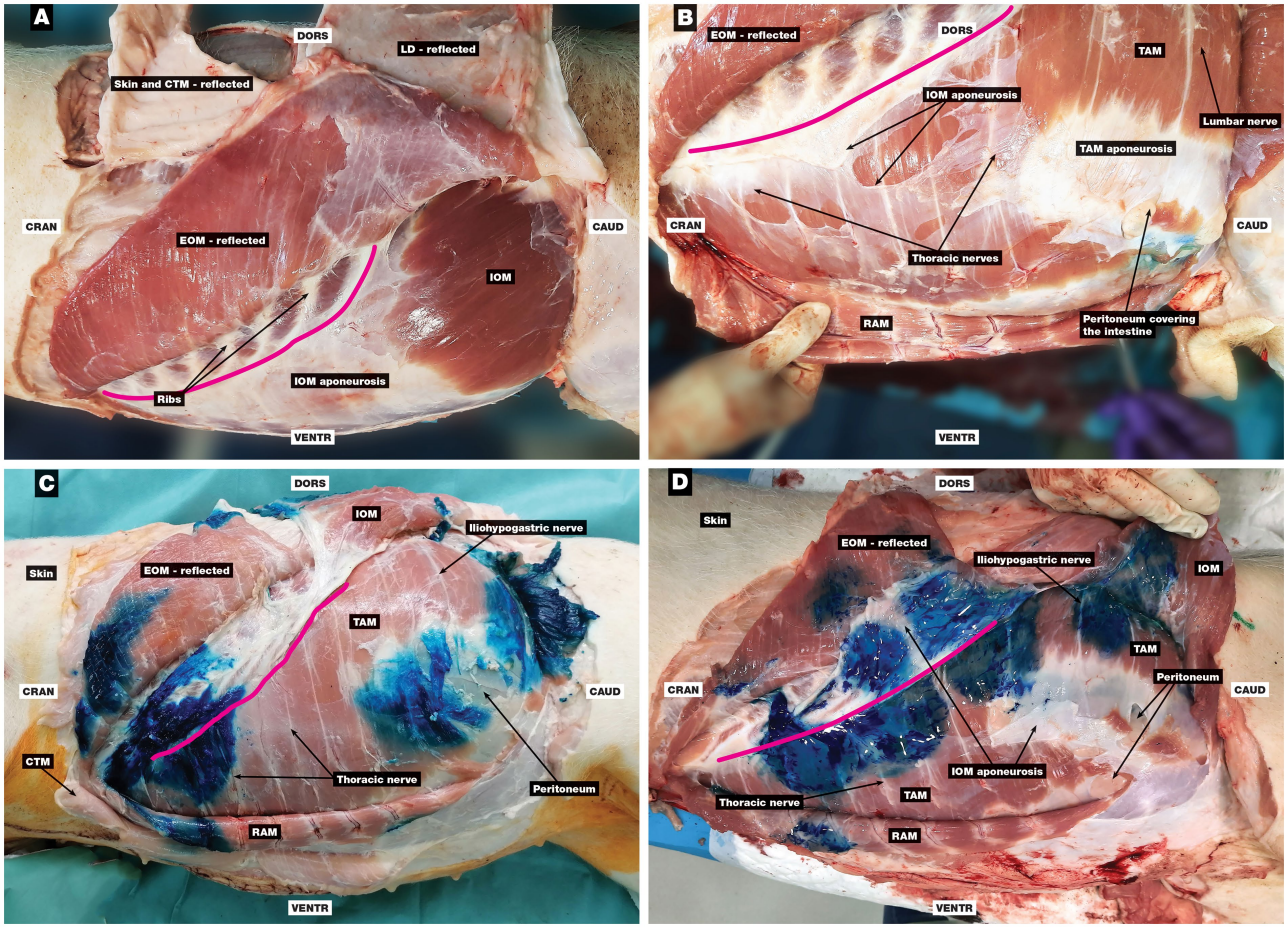
Anatomical dissection of the abdominal wall and evaluation of the spread of MB solution. **(A)** The EOM was reflected, and the IOM with aponeurosis and costal arch can be seen, **(B)** the EOM and IOM were reflected, and the RAM, TAM with its aponeurosis, and thoracic and lumbar nerves can be seen, **(C)** the three-point TAP technique: the EOM and IOM were reflected, and the RAM, TAM, and several stained thoracic and lumbar nerves can be seen within three separate staining areas of MB solution, **(D)** the four-point TAP technique: the EOM, IOM, and its aponeurosis were reflected, and the RAM, TAM with its aponeurosis, and stained thoracic and lumbar nerves can be seen; four staining areas can be observed, with the first two cranial staining areas intertwined. CAUD, caudal; CRAN, cranial; CTM, cutaneous trunci muscle; DORS, dorsal; EOM, external oblique abdominis muscle; IOM, internal oblique abdominis muscle; LD, latissimus dorsi muscle; RAM, rectus abdominis muscle; TAM, transversus abdominis muscle; VENTR, ventral; pink line, costal arch.

The EOM was mostly muscular with the aponeurosis running towards the ventral midline and the inguinal region. In contrast, the muscular part of the IOM was small and limited to the paralumbar region of the middle abdomen with an extensive aponeurosis passing cranioventrally to the costal arch and ventrally to the linea alba and umbilical region (Figure 6A). The aponeurosis of the IOM was very thin and, in some places, due to the interchange of aponeurotic fibres inherently connected to the aponeuroses of the EOM (ventrally towards the linea alba) and the TAM (cranially) or to the peritoneum (ventrocaudally towards the inguinal region). The TAM was mostly muscular with the aponeurosis oriented ventrally at the lateral abdominal region. The RAM was muscular with clearly visible tendinous intersections with vessels and nerves (Figure 6B).

Compared to the TAP technique in dogs, the positioning of the needle in the interfascial plane between the IOM aponeurosis and the TAM was challenging. No typical resistance was observed during perforation of the fascia, which made localization of the needle in the interfascial plane even more difficult (results of TAP injections are not presented).

### 3.2 Phase II

Two pigs had 16 ribs, and one had 15 ribs. In all six hemiabdomens, three clearly defined stained areas were observed, corresponding to the three-point TAP injections. In two hemiabdomens, MB solution was successfully injected between the IOM and TAM at all three points (Figure 6C). In the other four hemiabdomens, injections at the first and the third injection point were successfully applied between the IOM and TAM on the first attempt. At the second injection point, MB solution was falsely injected between the EOM and IOM in two hemiabdomens, while in the other two, it was administered correctly between the IOM and TAM after some repositioning of the needle. No signs of perforation of the internal fascia (transverse fascia) and parietal peritoneum were detected, as there was no intraabdominal distribution of the dye.

The number of positively/negatively stained nerves is presented in Table 1. The dye stains did not intertwine, and some nerves were not captured with one of the three injection locations. The positive staining was most successful at the seventh-last and sixth-last thoracic nerve (4 out of 6) and at L1 and L2 (4 and 6 out of 6, respectively).

**Table 1:** The number of stained nerves per animal in the phase II and III.

Animal	TAP block	Hemi-abdomen side	Thoracic nerves					Lumbar nerves						No. of stained nerves per animal
			8th-last	7th-last	6th-last	5th-last	4th-last	3rd-last	2nd-last	last	L1	L2	L3	
Cadaver 1	3-point	L	/	Y	N	N	Y	Y	Y	N	N	Y	N	5/10
		R	/	N	Y	N	N	N	N	Y	Y	Y	N	4/10
Cadaver 2		L	/	Y	Y	N	N	N	Y	Y	N	Y	Y	6/10
		R	/	Y	N	N	N	N	N	N	Y	Y	Y	4/10
Cadaver 3		L	/	Y	Y	Y	Y	N	Y	Y	Y	Y	N	8/10
		R	/	N	Y	Y	Y	N	N	N	Y	Y	N	5/10
Positively stained (>1 cm)			/	4/6	4/6	2/6	3/6	1/6	3/6	3/6	4/6	6/6	2/6	5.3/10 (53%)
Anaesthetized 1	4-point	L	N	N	Y	Y	Y	Y	Y	Y	Y	N	N	7/11
		R	N	N	Y	Y	Y	Y	Y	Y	Y	N	N	7/11
Anaesthetized 2		L	N	Y	Y	Y	Y	Y	N	Y	Y	Y	N	8/11
		R	N	Y	Y	Y	Y	Y	Y	Y	Y	Y	N	9/11
Anaesthetized 3		L	N	Y	Y	Y	Y	Y	N	Y	Y	N	N	7/11
		R	N	Y	Y	Y	Y	Y	N	Y	Y	N	N	7/11
Anaesthetized 4		L	Y	Y	Y	Y	Y	N	Y	Y	Y	N	N	8/11
		R	N	Y	Y	Y	Y	Y	Y	Y	Y	Y	N	9/11
Positively stained (>1 cm)			1/8	6/8	8/8	8/8	8/8	7/8	5/8	8/8	8/8	3/8	0/8	7.8/11 (71%)

TAP, Transversus abdominis plane; Y, stained nerve; N, unstained nerve; L1, L2, L3, lumbar nerves; L, left; R, right; /, not analysed.

**Figure 7:**
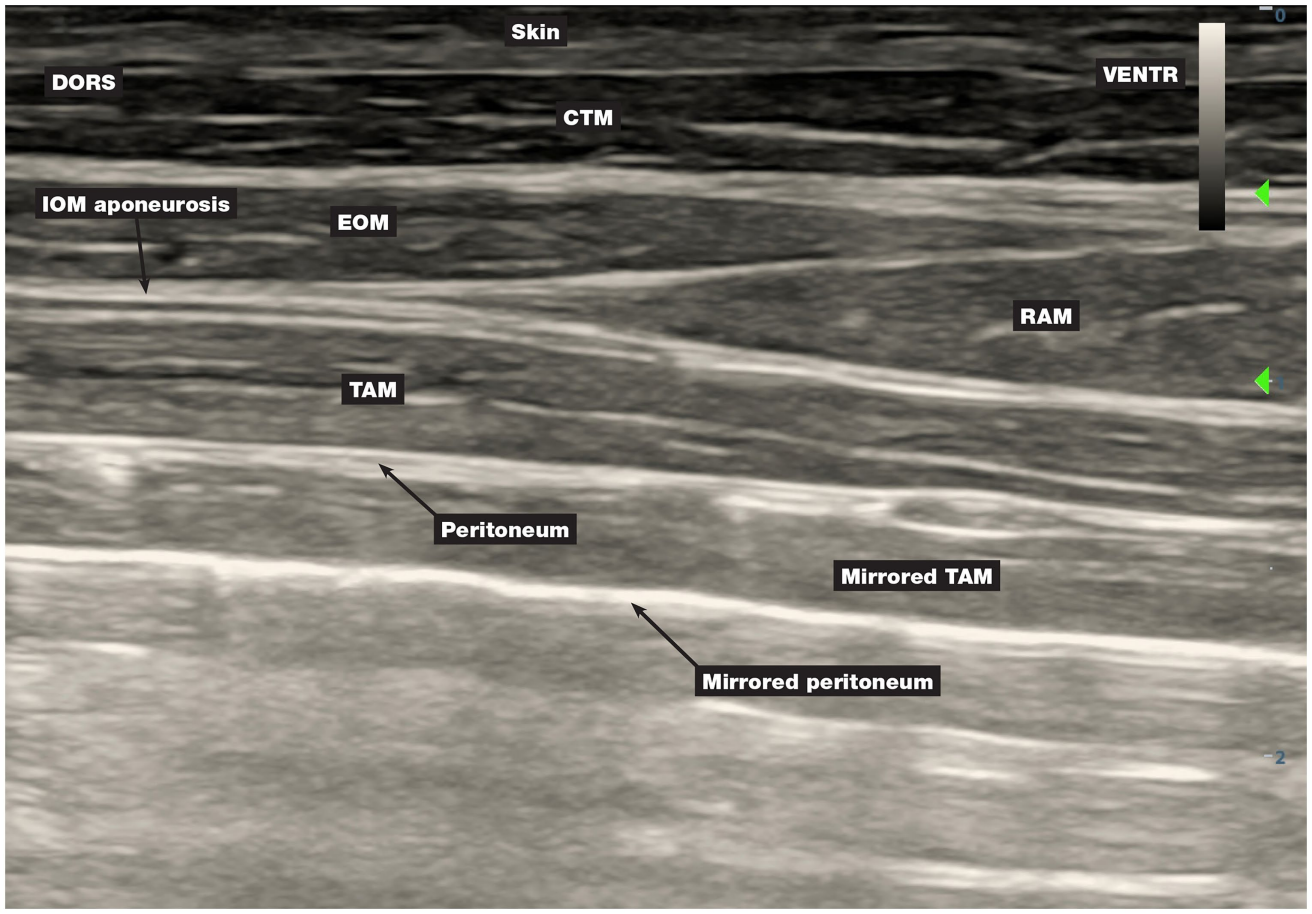
The mirroring artefact. The air that entered the abdominal cavity during abdominal surgery impeded the transmission and normal reflection of the ultrasound beam beyond the peritoneum. CTM, cutaneous trunci muscle; DORS, dorsal; EOM, external oblique abdominis muscle; IOM, internal oblique abdominis muscle; RAM, rectus abdominis muscle; TAM, transversus abdominis muscle; VENTR, ventral.

The mirroring artefact (Figure 7) has been observed in all pigs.

### 3.3 Phase III

All pigs had 15 ribs. In all eight hemiabdomens, MB solution was successfully injected between the IOM and TAM at all four injection points. No perforation of the internal fascia and peritoneum or intraabdominal distribution of the dye were observed.

Certain dye stains intertwined, which increased the number of positively stained nerves. In one animal, four stains (each for an individual injection point) were observed in both hemiabdomens. In five hemiabdomens (of three animals), only three stains were observed because they intertwined at the first and the second injection points (Figure 6D). In one hemiabdomen, two large stains were present, corresponding to the intertwining of the first with the second injection point and the intertwining of the third and fourth injection points. The number of stained nerves is presented in Table 1.

## 4 Discussion

This study describes the development of a four-point TAP technique targeting the somatosensory nerves of the cranial and mid-abdominal wall in grower pigs weighing 40–45 kg. This technique can be used for intraoperative desensitisation and postoperative analgesia of the abdominal wall in the area of the epigastrium and umbilicus.

In humans, the difference in the distribution of the dye between cadavers and patients has been demonstrated. The better distribution of local anaesthetic in cadavers was attributed to autolysis, and the extent of distribution was possibly related to time since death and ambient temperature (20). For this reason, anaesthetized pigs were used instead of cadavers in the final phase of this study in order to obtain more meaningful results on the extent of dye distribution.

Based on the dye distribution with previously described TAP injection techniques in pig cadavers (11, 12), desensitisation of a sufficient extent of the cranial part of the abdominal wall would not be achieved for surgeries requiring incisions up to the xyphoid process. The most cranial nerve stained with a two- and three-point TAP technique using 0.3 mL/kg of dye per hemiabdomen was the fifth-last thoracic nerve (12). Using a two-point TAP technique and 0.3 mL of dye per injection point, the fourth-last thoracic nerve was the most cranial nerve stained (11). In contrast, with the four-point TAP technique using 0.2 mL/kg of dye per injection point (0.8 mL/kg per hemiabdomen) in this study, the most cranial nerve positively stained was the eighth-last thoracic nerve, although in only one hemiabdomen. The seventh-last thoracic nerve was positively stained in six out of eight hemiabdomens, while the fifth-last thoracic nerve was positively stained in all cases. The injection points were closer together in the four-point TAP technique than in the three-point TAP technique, resulting in more positively stained nerves and the intertwining of certain adjacent dye stains with former technique. Since modern breeds of pigs have 14 to 16 thoracic vertebrae, we believe that a four-point TAP technique could provide better desensitisation of the cranial abdominal wall in pigs compared to the three-point TAP technique. Using the four-point TAP technique, the L2 nerve was stained with little success (3/8), and the L3 nerve was not stained at all. If the volume and concentration of the local anaesthetic allowed for it, a higher volume for the most caudal injection would probably result in a more caudal spread of dye.

The location of the interfascial plane at the first three application sites in phase III was challenging compared to the TAP technique in dogs, as it was difficult to distinguish the IOM aponeurosis from the TAM fascia. If the aponeurosis was not perforated and the dye was injected between the IOM aponeurosis and the EOM, the MB did not stain the nerves. To avoid false application, we used a technique involving the penetration of the TAM fascia and then carefully moving the needle back just above it (Figure 4B) while checking the position of the tip of the needle with the application of 1 mL of MB solution. This technique improved the success of needle positioning. The improved performance and increased experience of both anaesthetists may have also contributed to the higher percentage of positively stained nerves with the four-point TAP technique than with the three-point technique (21).

During phase I, several problems with the interpretation of US images and proper positioning of the needle were encountered and were related to *post-mortem* changes. Autolysis and putrefaction initiate immediately after death and change tissue consistency (22). No typical resistance was noted when the needle was advanced through the fascia of the animals that died a few days ago, which is consistent with other reports (23–25). In view of the difficulty in positioning the needle between the IOM aponeurosis and the TAM, the authors recommend the use of fresh cadavers for training purposes.

In phase II, a mirroring artefact (Figure 7) was observed, similar to that described in dogs in which TAP blocks were performed postoperatively (26). In phase II, organs from the abdominal cavity were explanted for the purposes of the primary study, the abdominal cavity was then sutured and the TAP injections were carried out. The air that entered the abdominal cavity during organ removal impeded the transmission and normal reflection of the US beam beyond the peritoneum, causing the mirroring artefact. The mirroring artefact is a type of reverberation artefact produced when the US beam encounters an interface that is capable of acting as a mirror (peritoneum/gas interface in this case) and is not reflected directly back to the transducer (27). Failure to recognise the mirroring artefact could lead to misidentification of structures and application of anaesthetic between the false layers. The mirroring artefact in phase II made it difficult to localise the corresponding interfascial plane, and the MB solution was incorrectly injected between the EOM and IOM at the second injection point in two hemiabdomens which was not the case in phase III.

This study has some limitations. First, the number of experimental pigs in phase II and III was limited by the primary study. Second, due to the requirements of the primary study, the abdominal organs were explanted, the abdominal wall was sutured, and pigs were euthanized prior to TAP injection in phase II. The presence of air in the abdominal cavity caused the mirroring artefact that complicated the interpretation of the US image and resulted in injection of the MB solution into false interfascial plane. Third, although the phase II TAP injections were performed shortly after euthanasia, some postmortem changes may have already begun and altered tissue properties. In addition, the lack of blood flow, tissue movement, and changes in cavity pressure may have affected the distribution of MB solution (28). Finally, there is the possibility that the MB solution has not spread in the same way as the local anaesthetic in the interfascial plane.

## 5 Conclusion

The four-point TAP injection technique with 0.2 mL/kg MB solution per application site enabled successful staining of the last seven thoracic nerves and the first lumbar nerve, whereby the L2 nerve was stained with less than 50%. This technique could allow intraoperative desensitisation and postoperative analgesia of the abdominal wall for surgery in the epigastric and umbilical region of pigs with 15 to 16 thoracic vertebrae weighing 40–45 kg. Further studies in live animals are needed to evaluate the clinical applicability and efficacy of desensitisation with the four-point TAP block technique.

## Data Availability

The original contributions presented in the study are included in the article/supplementary material, further inquiries can be directed to the corresponding author.
